# Risk factors for osteoporosis in chronic schizophrenia on long-term treatment with antipsychotics: a cross-sectional study

**DOI:** 10.1186/s12888-023-04951-1

**Published:** 2023-06-21

**Authors:** Furu Liu, Ying Wu, Jindong Chen, Tianxiang Zou, Yue Qin, Ziwei Teng, Yuhan Su, Renrong Wu, Jing Huang

**Affiliations:** 1 Department of Psychiatry, National Clinical Research Center for Mental Disorders, National Center for Mental Disorders, National Technology Institute on Mental Disorders, The Second Xiangya Hospital of Central, South University, 410011 Changsha, Hunan China; 2 Department of Intensive Care Unit, The Second Xiangya Hospital of Central South University, 410011 Changsha, Hunan China

**Keywords:** Bone mineral density, Schizophrenia, Osteoporosis, Gender, Risk factor

## Abstract

**Background:**

Little is known about the laboratory variable risks with bone mineral density (BMD) in patients with schizophrenia. This study was designed to fully investigate the related risk factors for decreased BMD in schizophrenia, as well as evaluate the gender difference of BMD.

**Method:**

The BMD of the forearm of 211 patients (males/females = 140/71) who met the diagnostic criteria for DSM-5 schizophrenia was measured by dual-energy X-ray absorptiometry. Basic demographic information, clinical assessments, and laboratory variables (regarding nutrition, hormones, metabolism, and inflammatory markers) were comprehensively collected.

**Results:**

Among 211 subjects, seventy-four (35%) patients had low BMD. Males had a significantly lower BMD T-score than females (*P =* 0.002). Multiple regression analyses showed that the independent risks with low BMD were lower folate, glycosylated hemoglobin levels, higher age, serum ferritin, and follicle-stimulating hormone (FSH) levels. In female patients, the BMD was mainly associated with age and serum hormones (FSH and testosterone), while the BMD of male patients was primarily related to age, microelements (serum ferritin and 25-OH-VD), and parathyroid hormone.

**Conclusion:**

Our study found several meaningful correlations between osteoporosis and schizophrenia, especially regarding laboratory measures, which may provide new clues to identifying or preventing osteoporosis in clinical patients.

**Supplementary Information:**

The online version contains supplementary material available at 10.1186/s12888-023-04951-1.

## Introduction

Osteoporosis is a global health problem attracted much concern in past decades. Approximately 200 million individuals worldwide are affected by osteoporosis, which results in 8.9 million fractures annually [1]. Osteoporosis is characterized by low bone mineral density (BMD) and progressive degradation of bone microstructure, with consequently increased fragility and fracture risks [2]. The reduced bone density was associated with high morbidity for fracture, especially of the hip, spine, and wrist [3]. The increased incidence of osteoporotic fractures was a significant cause of mortality [4], as hip fractures result in a 10–20% increase in mortality risk within 1 year in hospital [5]. Osteoporosis-related fractures may result in a variety of physical and psychological consequences, including pain, depressive symptom, functional impairment, or disability, which lead to a high socioeconomic burden on individuals, families, and societies.

Schizophrenia is a serious and relapsing mental disease that requires long-term antipsychotic treatment. Patients with chronic schizophrenia are prone to comorbidity with metabolic disorders, in which abnormal glucose and lipid metabolisms have been widely explored, while bone metabolism was poorly understood. Several recent studies have reported that the incidence of low BMD was significantly higher in patients with schizophrenia than in the generational population [6–8]. A cohort study of 10-year follow-ups revealed approximately double the risks of hip and vertebral fractures in patients with schizophrenia compared with the control group [9]. This issue increased 54% of the risk of mortality [6], resulting in a heavy burden of disease. In addition, gender difference has been reported in BMD of schizophrenia [10, 11]. A report of 965 chronic patients with schizophrenia pointed out that 9.2% of males and 21% of females have osteoporosis[12], while another meta-analysis study enrolled 3,038 patients with schizophrenia indicated that males tended to have low BMD than females [13]. These inconsistent findings indicate that gender differences in bone mineral density in patients with schizophrenia deserve more attention.

Schizophrenia is associated with decreased bone mineral density. However, the underlying mechanism for the increased risk of osteoporosis in patients with schizophrenia remains unclear. Over the past few years, research has mainly focused on the association between antipsychotic-induced hyperprolactinemia and BMD. Results showed that hyperprolactinemia can lead to the reduced secretion of sex hormones, like estrogen and androgen, which deficiencies can imbalance the homeostasis of osteogenesis [14]. Patients suffering from schizophrenia were at risk of many factors including inadequate physical activity, smoking and drinking habits, poor nutrition, and vulnerability to metabolic syndrome [15]. Recent evidence suggested that biochemical indexes (like blood lipid [16], serum hemoglobin (Hb) [17], and serum ferritin (SF) [18]) and liver-related disease [19] were also associated with BMD in general adults. A study by Zhang et al. [20] found that accelerated bone resorption was significantly related to glucolipid metabolism and insulin resistance in patients with schizophrenia, especially the level of triglyceride (TG), high-density lipoprotein cholesterol (HDL), and glucose (GLU). However, to date, the research on how these factors interact to affect bone density in patients with schizophrenia is still limited. Identifying the relationship between biochemical indicators (including nutrition, endocrine hormone, metabolism, and inflammation) and BMD may promote to disclosure of the mechanism of osteoporosis in patients with schizophrenia and provide information for clinical prevention and management. Therefore, in the present study, we aimed to explore the potential risk factors for decreased BMD with schizophrenia, as well as evaluate the gender difference in schizophrenia.

## Methods

### Designs and participants

This cross-section study was carried out in a chronic inpatient ward and enrolled a total of 211 patients with schizophrenia. All recruited patients met the following inclusion criteria: (1) age 18–75 years old and Han Chinese; (2) diagnosed with schizophrenia according to the Diagnostic and Statistical Manual of Mental Disorders, Fifth Edition (DSM-5); (3) had stable antipsychotics medication for at least 6 months; (4) no other severe cardiovascular, renal, central nerve or gonadal diseases that may affect bone metabolisms; (5) no other psychotic diagnoses in addition to schizophrenia, such as substance dependence and abuse; and (6) not pregnant or breastfeeding. Meals are provided by the hospital canteen during hospitalization, all patients had a similar diet condition and regular physical activity after admission.

The study protocol was approved by the Human Research and Ethics Committee of the Second Xiangya Hospital, Central South University, China (2016YFC1306900). The investigation was carried out following the Declaration of Helsinki. After the study procedure had been completely described to all subjects, they signed written informed consent. Any procedure related to this study was performed after obtaining informed consent.

According to the T-score, patients were assigned into a normal BMD group, osteopenia group, and osteoporosis group. Demographic, clinical, and laboratory indices were assessed to identify the factors associated with low BMD in schizophrenia. Besides, patients were later grouped based on gender for the purpose to evaluate the gender difference in BMD and relative factors.

### Clinical information

Basic demographic and clinical information was collected via the questionnaire that was specifically designed for this study. The general information which may relate to BMD was obtained by well-trained researchers, including age, gender, date of birth, education, marital status, vocation, age of onset, course, smoking and alcohol history, previous drug history, current drug admission list and equivalent chlorpromazine (CPZ) dose of current antipsychotics, family mental disorder and metabolism disease histories, medical conditions, weight, and body mass index (BMI).

Clinical symptoms were rated using the SANS (Scale for the Assessment of Negative Symptoms) and SAPS (Scale for the Assessment of Positive Symptoms).

### Laboratory assessment

Blood samples were collected from all patients between 7 a.m. to 9 a.m. after fasting overnight. To investigate the related factors with BMD, using samples, we measured indicators of several aspects including nutrition conditions [ex. folate, vitamin B12 (Vit-B12), 25-OH-VD, Hb, SF, calcium (Ca)], endocrine hormones [ex. parathyroid hormone (PTH), free triiodothyronine (FT3),  estradiol (E2), testosterone (T), follicle-stimulating hormone (FSH), luteinizing hormone (LH), procalcitonin (PRL)], and metabolism [ex. serum GLU, total TG, HDL, glycosylated hemoglobin (HbA1c), CRE (creatinine)]. In addition, other clinical lab indexes related to inflammation such as white blood cell accounting (WBC), platelet (PLT) count, and C-reactive protein (CRP) were measured in all patients.

### BMD measurement

In the present study, BMD was measured with dual-energy X‐ray absorptiometry (DEXA) (Hologic, USA) at the forearm. T-score values were used to compare the bone mineral density and standardized bone mass of groups in our current study. We used the World Health Organization criteria defining osteopenia as a BMD of more than 1 but less than 2.5 standard deviations (SD) below the mean for young adults (T scores < − 1 and > − 2.5) and osteoporosis as a BMD of 2.5 SD or more below the young adult mean (T score ≤ − 2.5). Normal BMD is defined as a BMD ≥ 1 SD above the mean (T score ≥ − 1). Low bone mass (including osteopenia and osteoporosis) is defined as a BMD T score of < − 1.

### Statistical analysis

Data were analyzed with SPSS 25.0 (2017) and results are presented as percentages or mean ± standard deviation. Between groups, differences were measured by ANOVA for continuous variables and the chi-squared test for categorical variables. Independent T-test and the rank-sum test was used between the male and female group. The relationships between variables and bone mass were evaluated using Pearson’s correlation or Spearman correlation analyses. Factors associated with low BMD T-score in univariate analyses (*P* < 0.05) and known risk factors (like age, calcium, and vitamin D) were entered collectively into the multivariate analysis. Multiple stepwise regression analyses were used to identify predictors that correlated with decreased BMD T-score. A *P*-value, of 0.05 was considered significant.

## Results

### Demographic and clinical characteristics of patients

As shown in Table 1, a total of 211 patients (140 males and 71 females) were enrolled. The average age of the patients was 48.6 (SD 12.3) years old and the duration of illness was 21.8 (SD 11.4) years. The mean BMI was 24.8 (SD 4.8) kg/m^2^ and 52% of the patients were identified as overweight. The average BMD T-score was − 0.5 (SD 1.6).

**Table 1:** Comparison of demographic and clinical characteristics of patients with schizophrenia

Characteristic	Total	Normal^1^	Osteopenia^2^	Osteoporosis^3^	*P*-value	Post Hoc
	(N = 211)	(N = 137)	(N = 42)	(N = 32)		
Age (years)	48.6 (12.3)	46.1 (10.7)	49 (13.5)	58.8 (11.0)	< 0.001	1^**^,2^**^<3
Gender (M/F)	140/71	84/53	32/10	24/8	0.139	
Marital status					0.053	
Unmarried	122	79	28	15		
Have a spouse	39	27	9	3		
Divorced	44	28	5	11		
Death of a spouse	6	3	0	3		
Education (years)	7.1 (3.6)	7.6 (3.4)	6.4 (4.1)	5.6 (3.3)	0.017	1 > 3^*^
Body weight (kg)	67.4 (12.6)	68.7 (13.1)	66.1 (11.7)	63.5 (10.5)	0.084	
BMI (kg/m2)	24.8 (4.2)	25.3 (4.3)	24.1 (4.1)	24.7 (4.2)	0.022	1 > 3^*^
BMD	0.6 (0.1)	0.6 (0.1)	0.5 (0.1)	0.4 (0.1)	< 0.001	1 > 2^**^>3^**^
T-score	-0.5 (1.6)	0.4 (1.1)	-1.7 (0.4)	-3.0 (1.0)	< 0.001	1 > 2^**^>3^**^
Duration of illness (months)	261.6 (136.8)	251.4 (127.4)	259.1 (145.3)	313.5 (159.1)	0.019	1 < 3^*^
Smoking (Y/N)^a^	54/150	32/100	11/30	11/20	0.560	
Drinking (Y/N)^a^	18/184	14/117	3/38	1/28	0.451	
Family history of mental illness (Y/N)	47/164	23/114	14/28	10/22	0.037	1 < 2^*^,3^*^
Family history of metabolic syndrome (Y/N)	4/207	3/134	1/41	0/32	0.685	
Antipsychotics^b^					5.204	0.246
PRL-increasing-Aps	10	5	1	4		
Non-PRL-increasing-Aps	54	36	11	17		
Mixed	147	96	30	21		
Antipsychotic CPZ-dose (g/day)	0.7 (0.4)	0.7 (0.3)	0.6 (0.5)	0.6 (0.4)	0.409	
SANS score	44.6 (15.1)	43 (14.3)	41.3 (15.6)	51.3 (17.3)	0.010	1^*^,2^**^<3
SAPS score	10.7 (8.1)	11.5 (8.1)	9.4 (8.1)	8.8 (8.2)	0.218	

Note: ^a^ including missing data. (* *P* < 0.05, ** *P <* 0.001). ^b^ No significant differences in polypharmacy status among patients. PRL-increasing-Aps: antipsychotics related with hyperprolactinemia or elevated prolactin levels including fluphenazine, risperidone, haloperidol, amisulpride, sulpiride, and paliperidone. Non-PRL-increasing-Aps: including clozapine, mirtazapine, olanzapine, quetiapine, and aripiprazole. Mixed: using both PRL-increasing-Aps and non PRL-increasing-Aps. Group codes in post hoc: 1, Normal; 2, Osteopenia; 3, Osteoporosis

Among 211 patients, 74 (35.1%) had low BMD T-score, including 42 (19.9%) with osteopenia, and 32 (15.2%) with osteoporosis. 64% of patients over 50 years old had bone loss, conversely, only 21.9% of patients younger than 50 years old. There was no significance between the three groups in gender, marriage status, body weight, daily antipsychotic CPZ-equivalent dose, smoking, and drinking history. However, compared to patients without osteoporosis, patients with osteoporosis were older and had higher SANS scores. Moreover, patients with osteoporosis had lower education years and BMI than patients with normal T-scores. The percentage of patients with a family history of mental disorders in the osteopenia and osteoporosis groups was higher than those in the normal T-score group. Table 1 shows the demographic and clinical characteristics of the group according to T-scores.

### Males had low BMD T-sores than females

Comparisons between males and females in clinical and demographic characteristics were listed in Supplement Table 1. The rates of low BMD (osteopenia and osteoporosis) among males were 40%, while among females was 25%. Males had a significantly lower BMD T-score than females (t=-3.20, *P =* 0.002). The average body weight was significantly higher in males compared to those in females (t = 4.71, *P <* 0.001). However, there were no statistically significant differences in BMI, education years, duration of illness, smoking and drinking habits, family history of mental illness and metabolic syndrome, SANS and SAPS scores, and daily antipsychotic CPZ-equivalent dose.

### The biochemical difference among patients

Laboratory evaluation in several aspects were detailed in Table 2. Comparisons of the major variable between males and females were presented in Table 3.

**Table 2:** Major biochemical characteristics of the study patients

Variables	Normal^1^	Osteopenia^2^	Osteoporosis^3^	F/Z	*P*-value	Post Hoc
	(N = 137)	(N = 42)	(N = 32)			
**Nutrition**						
Folate (ng/ml)	7.6 (4.5)	7.4 (4.4)	5.9 (2.7)	1.51	0.225	
Vit-B12 (pg/ml)	379.2 (193.0)	490.8 (336.8)	402.5 (290.6)	2.40	0.094	
25-OH-VD (ng/ml)	29.7 (8.4)	28.9 (8.2)	26.8 (6.5)	1.63	0.190	
Ca (mmol/L)	2.2 (0.1)	2.3 (0.1)	2.2 (0.1)	0.29	0.746	
SF (µg/L)	106.2 (81.6)	180.3 (90.9)	139.6 (106.0)	8.61	**< 0.001**	1 < 2^**^
Hb (g/L)	136.4 (16.1)	142.9 (18.7)	138.1 (16.3)	2.39	0.094	
**Hormones**						
PTH (pg/ml)	46.4 (25.7)	50.5 (30.9)	49.1 (32.2)	0.40	0.669	
FT3 (pmol/L)	4.4 (0.8)	4.4 (0.9)	4.2 (1.0)	0.33	0.722	
FSH (IU/L)	11.6 (16.7)	15.2 (22.7)	20.7 (28.7)	2.76	0.066	
LH (IU/L)	6.4 (7.1)	8.2 (9.5)	10.4 (11.5)	2.90	0.057	
E2 (pmol/L)	137.1 (142.1)	139.9 (254.9)	123.7 (168.6)	0.06	0.946	
T (nmol/l)	9.6 (9.1)	12 (9.2)	11.9 (8.6)	1.80	0.169	
PRL (ng/ml)	36 (31.4)	28.3 (22.3)	30.9 (25.7)	1.30	0.275	
**Metabolism**						
HbA1c (%)	5.7 (0.9)	5.7 (0.9)	5.3 (0.7)	3.76	**0.025**	1 < 3^*^
CRE (µmol/L)	63.3 (16.6)	65.7 (14.7)	69.8 (15.3)	2.21	0.113	
GLU (mmol/L)	5.5 (1.5)	5.4 (1.2)	4.9 (0.6)	2.52	0.083	
TG (mmol/L)	1.5 (0.8)	1.4 (0.7)	1.4 (0.7)	0.15	0.858	
HDL (mmol/L)	1.2 (0.3)	1.1 (0.3)	1 (0.2)	2.15	0.120	
**Inflammation**						
WBC (*10^9/L)	7.1 (2.2)	6.6 (2.7)	6.7 (2.4)	1.15	0.318	
PLT (*10^9/L)	233.5 (63.8)	220.9 (58.5)	216.9 (72.2)	1.23	0.295	
CRP (µg/L)	3.3 (6.3)	4.4 (6.5)	1.9 (1.3)	0.73	0.486	

Note: ^a^ including missing data. (* *P <* 0.05, ** *P <* 0.001)

Group codes in post hoc: ^1^ Normal; ^2^ Osteopenia; ^3^ Osteoporosis

**Table 3:** Gender-based comparisons of biochemical characteristics in patients

Variables	Male	Female	T/Z	*P*-value
	(N = 140)	(N = 71)		
**Nutrition**				
Folate (ng/ml)	5.9 (3.3)	10 (4.6)	−5.62	**< 0.001**
Vit-B12 (pg/ml)	400.6 (255.7)	408.4 (214.0)	0.04	0.847
25-OH-VD (ng/ml)	29.5 (8.0)	28.5 (8.5)	0.71	0.400
Ca (mmol/L)	2.2 (0.1)	2.3 (0.1)	0.63	0.427
SF (µg/L)	147.1 (90.1)	89.1 (82)	16.58	**< 0.001**
Hb (g/L)	144 (15.6)	125.9 (11.7)	−7.62	**< 0.001**
**Hormones**				
FT3 (pmol/L)	4.4 (0.9)	4.3 (0.8)	0.73	0.394
PTH (pg/ml)	47 (24.9)	49.5 (33.1)	0.37	0.543
FSH (IU/L)	6.3 (4.1)	28.8 (29.6)	−5.04	**< 0.001**
LH (IU/L)	4.7 (2.4)	12.5 (12.7)	−4.40	**< 0.001**
E2 (pmol/L)	87.7 (53.9)	244.1 (269.1)	−3.19	**< 0.001**
T (nmol/l)	15.2 (7.7)	1.3 (1.5)	−11.66	**< 0.001**
PRL (ng/ml)	29.5 (23.8)	42.1 (35.8)	9.26	**0.003**
**Metabolism**				
HbA1c (%)	5.4 (0.7)	6 (0.9)	23.47	**< 0.001**
GLU (mmol/L)	5.2 (1.1)	5.7 (1.7)	5.73	**0.048**
CRE (µmol/L)	70.9 (14.9)	52.6 (10.8)	−8.31	**< 0.001**
TG (mmol/L)	1.6 (0.8)	1.3 (0.6)	5.16	**0.024**
HDL (mmol/L)	1 (0.2)	1.3 (0.3)	6.80	**< 0.001**
**Inflammation**				
WBC(*10^9/L)	7 (2.2)	6.8 (2.7)	0.43	0.515
PLT (*10^9/L)	220.6 (60.1)	243.8 (69.3)	6.29	**0.013**
CRP (µg/L)	3.4 (6.6)	3.1 (3.9)	0.07	0.798

#### Nutrition factors

As shown in Table 2, the SF level of the osteopenia was significantly higher than those of the normal BMD group (*P =* 0.034). No significant difference was found in folate, Vit-B12, 25-OH-VD, calcium, and Hb levels between the three groups. However, according to the normal reference values, the average value of folate was below the normal range (10.4–42.4 mmol/L). As shown in Table 3, compared with the female group, a statistically significant higher SF level, higher Hb level, and lower folate level were seen in the male group. There was no significant difference in Vit-B12, 25-OH-VD, and calcium levels between the male and female groups.

#### Endocrine hormones

No significant differences in thyroid function and sex hormones indicator levels were found in the osteopenia or osteoporosis group as compared to the normal BMD group. Moreover, the average value of PRL was higher than the normal range (5-25ng/ml). There were similar levels of FT3 and PTH levels between males and females. However, the male group had significantly lower PRL levels as compared with the female group (z = 61.88, *P =* 0.003). Males had significantly higher T levels, lower FSH levels, LH levels, and E2 levels than those females.

#### Metabolism-related variables

Regarding all evaluated metabolism-related indicators, only the HbA1c level of patients with osteoporosis was lower than those in patients with normal BMD T-score (*P <* 0.05). Males had significantly lower GLU (t = 5.73, *P =* 0.048), and HDL (t = 61.89, *P <* 0.001) levels than those in females. Moreover, males had significantly higher CRE levels (z = 84.53, *P <* 0.001) and TG levels (t = 5.16, *P =* 0.024) as compared to females.

#### Inflammation

Table 2 also shows the blood inflammation markers for the three groups. There was no significant difference in the accounting of WBC, PLT, and CRP levels between the three groups. Males had significantly lower PLT levels (t = 6.29, *P* = 0.048) than those females. No difference was found in the accounting of WBC and CRP levels between males and females groups.

### Factors associated with BMD T-score in patients

Correlation analyses were performed between the major variables, including demographic variables, clinical characteristics, and biochemical variables, which may affect BMD T-score (Supplement Table 2). The value of BMD T-score was positively correlated with gender (male = 1; female = 2; r = 0.233, *P =* 0.001), folate level (r = 0.233, *P =* 0.001), HLD level (r = 0.203, *P =* 0.010), HbA1c level (r = 0.139, *P =* 0.044) and SANS score (r = 0.155, *P =* 0.025). Negative correlations were found between BMD T-score and age (r=-0.343, *P <* 0.001), SF level (r=-0.267, *P =* 0.001), LH (r=-0.185, *P =* 0.007), T (r=-0.190, *P =* 0.006), GLU (r=-0.160, *P =* 0.021), and attention dysfunction score (r=-0.195, *P =* 0.005).

### Predictive factors of BMD T-score in patients

Multiple linear regression results showed that age (beta=-0.258, *P =* 0.001), folate (beta = 0.185, *P =* 0.016), SF level (beta=-0.283, *P* < 0.001), HbA1c level (beta = 0.186, *P =* 0.017), and FSH level (beta=-0.272, *P =* 0.001) were associated with BMD T-score (Adjusted R^2^ = 0.259, Table 4).

**Table 4:** Multiple linear regression analysis of predictive factors associated with the BMD T-score in schizophrenia

Variables	Unstandardized Coefficients		Standardized Coefficients	t	*P*	95% Confidence Interval for B
	B	SE.	Beta			
(Constant)	-0.456	0.963		-0.474	0.636	(-2.361, 1.449)
Age	-0.036	0.011	-0.258	-3.244	0.001	(-0.058, -0.014)
SF	-0.005	0.002	-0.283	-3.636	< 0.001	(-0.008, -0.002)
FSH	-0.020	0.006	-0.272	-3.369	0.001	(-0.032, -0.008)
Folate	0.070	0.029	0.185	2.436	0.016	(0.013, 0.127)
HbA1c	0.364	0.151	0.186	2.416	0.017	(0.066, 0.663)

Besides, multiple linear regression was also performed to identify the predictive factors of BMD T-score between male and female patients’ subgroups. In males, age (beta=-0.256, *P =* 0.007), SF levels (beta=-1.988, *P =* 0.050), serum 25, OH-VD levels (beta = 2.217, *P =* 0.029), and serum PTH levels (beta = 3.061, *P =* 0.003) was associated with the BMD T-score. (Adjusted R^2^ = 0.205, Supplement Table 3). In females, age (beta=-0.272, *P =* 0.028), serum FSH levels (beta=-0.321, *P =* 0.066), and serum T levels (beta = 0.205, *P =* 0.035) were associated with the BMD T-score. (Adjusted R^2^ = 0.426, Supplement Table 3).

## Discussion

This study was to fully explore the biochemical variables associated with decreased bone mass in patients with schizophrenia. Our results revealed several clinical and biochemical factors that were associated with loss of bone mineral density. The males were more likely to have decreased BMD than females, still, there were no significant differences in age, BMI, and other clinical characteristics.

The prevalence of decreased bone mineral density among patients with schizophrenia was 35.1% (osteopenia = 42 and osteoporosis = 32), which was higher than the prevalence reported 20.4% in a study of 285 community-dwelling patients with psychotic disorders [21] and lower than the prevalence of 65.3% in Cui et al.’s study involving 199 Chinese inpatients with schizophrenia [7]. This inconsistency is mainly due to the differences in the distribution of age and gender in the samples, as well patients from different settings. A previous meta analysis showed that the prevalence of low BMD higher in inpatient setting than outpatients [13]. Age was the most common cause of bone loss. Interestingly, our results showed that patients with schizophrenia aged more than 50 years old had 64% decreased BMD, this figure was higher than that of patients with schizophrenia in the overall age group (53.2%) [13]. A report suggested that differences in BMD between schizophrenic patients and controls were not significant until the 30s, whereas differences could be detected in the 40s or older [22]. Consistent with the above studies, age also was a significant risk factor for decreased BMD in both genders in the present study. Therefore, substantial concern should be paid to bone mass in patients with schizophrenia, especially in elderly patients.

In the present study, we found that decreased BMD T-score was significantly correlated with high levels of SF and low levels of folate after the multiple linear regression. Iron as an essential component of heme synthesis plays an important role in human health, and iron deficiency can lead to many pathologic consequences such as weakness and immune dysfunction [23]. Excess iron decreases osteoplastic activity and promotes bone resorption and osteogenic differentiation [24]. SF is a reliable measure of iron status, particularly iron overload [25]. The negative relationship between SF and BMD in our findings in patients with schizophrenia was consistent with previous studies [26–28]. A recent study indicated that SF appeared to be a better iron variable in relation to BMD when compared with iron intake and serum iron, and higher SF was associated with lower femoral neck and lumbar spine BMD after controlling the multiple covariates (i.e., age and race) [18]. Furthermore, although iron deficiency-related anemia causes osteoporosis, an interesting result of a large sample study in Korea illustrated that Hb levels had a significant inverse association with BMD [17]. These results may reveal the independent ways in which SF and hemoglobin influence BMD. However, the underlying mechanism requires explanation in future studies.

In addition, the folate deficiency of numerous patients in this sample was consistent with other studies [29–31]. Folate deficiency has been identified as a risk factor for schizophrenia, which affects fetal brain development and manifests as behavior and cognition impairment [32]. A previous review has described that folate and Vit-B12 deficiency were correlated with high levels of serum homocysteine [33], which elevated can increase the risk for fractures. However, only levels of serum folate positively related to BMD after regression analysis in our study. Cagnacci et al. [34] investigated whether the levels of homocysteine and critical coenzymes of homocysteine metabolism were related to BMD in 161 postmenopausal women, the results showed that folate, but not homocysteine or Vit-B12, was independently associated with BMD. Similarly, Holstein et al. [35] also reported that low serum folate and vitamin B6 concentrations, but not low serum vitamin B12 concentrations, were associated with an altered morphology in human bone. The mechanism of the association between folate and BMD is unclear. Research showed that as an effective free radical scavenger, folate can prevent DNA damage, inhibit oxidative stress, and reduce cell apoptosis [36]. Besides, folate may have direct effects on bone cells. A recent study found that folate attenuated bone loss and enhanced bone microstructure in high-fat diet mice, accompanied by the number of osteoclasts and adipocytes decreased [37]. Inadequate intake and inappropriate absorption of folate may contribute to low levels of serum folate. Thus, as an important nutrient for bone mineralization, whether prophylactic supplementation of folate can prevent the loss of bone density remains to be explored.

Beisdes, our study showed that patients with osteoporosis had higher SANS scores than patients with normal BMD and osteopenia. The negative behavioral patterns resulting from psychopathological symptoms (including low levels of physical activity, lack of sunshine exposure, and a sedentary lifestyle) may decrease bone density [38]. This finding reminds clinicians that should pay attention to life interventions for patients with chronic schizophrenia, and more time for outdoor activities may help to restore bone health and reduce the risk of BMD loss.

Interestingly, regarding the relationship between BMD and metabolism variables, we found that only HbA1c had statistically significant. Evidence from clinical and basic research illustrated a strong interaction between bone metabolism and glucose levels. A large prospective study showed that each 1% reduction in mean HbA1c was associated with a 37% reduction in risk for any microvascular complications related to diabetes [39]. Considering the relationship between HbA1c levels and bone fragility, optimal glycemic control may reduce the risk of fracture in patients with mental disorders.

Despite previous clinical studies indicating that high PRL levels were the risk factor for osteoporosis in patients with schizophrenia [40], we only found that BMD was negatively associated with LH and T levels in patients. Studies have shown that antipsychotic-induced hyperprolactinemia leads to decreased secretion of FSH and LH, in turn, which can lower the secretion of estrogen [15, 41]. Estrogen promotes osteogenesis and has an inhibitory effect on osteoclasts. In our results, for the long-term administration of antipsychotics, the average serum prolactin of our sample was beyond the normal value. The linear regression analysis showed that FSH levels were associated with BMD changes in patients with schizophrenia. A cross-sectional study including 699 Chinese women confirmed that the serum FSH and LH were significantly negative with the bone mass at different skeletal sites [42]. Elevated FSH levels are associated with loss of gonadal function during the menopausal transition [43]. Several epidemiological studies of perimenopausal women have shown that increases in the bone turnover index or BMD are most associated with increases in serum FSH, rather than E2, throughout the menopausal transition [44, 45]. Our findings in patients with schizophrenia were consistent with those results.

Furthermore, gender differences were observed in the prevalence of low bone mineral density in patients with schizophrenia. In line with previous studies [21, 46, 47], male patients were more susceptible to low BMD than female patients. In addition, a meta-analysis showed a higher prevalence of osteopenia and osteoporosis in males [13]. However, some studies have shown the opposite results. Jung et al. reported that in the general population, females presented a high rate of low BMD compared with males [48]. Many complex factors contribute to this discrepancy results, like types of antipsychotic treatment, physical conditions, and nutritional status. Regression analysis also revealed gender differences in factors affecting BMD. In women, osteoporosis is attributed primarily to the loss of gonadal steroids during the menopausal transition, especially FSH, LH, and T. However, in men, low BMD was associated with high SF levels, low serum 25, OH-VD levels, and low PTH levels. BMD in men appears to be less affected by hormones, the normal testicular function does decline in men with increasing age (> 70 years) [49]. Therefore, the gender difference in bone metabolism in patients with schizophrenia requires further investigation, which can help to provide personalized preventive measures for osteoporosis in patients with schizophrenia.

There are some limitations in this study. First, the sample size of this study was relatively small with an unbalanced proportion of sex. Therefore, a large size and gender-matched sample study need to be conducted to verify the findings. Second, the blood sample collection of female patients largely from different menstrual cycles will cause selection bias to a certain extent. Third, this was cross-sectional nature research, which requires a prospective and longitudinal design to elucidate the direct causal relationship between clinical variables and osteoporosis in patients with schizophrenia.

## Conclusion

In summary, our results provide further evidence of risk factors related the decreased BMD or osteoporosis in patients with schizophrenia, and it showed gender differences. Patients with low BMD tend to be older and have higher SF, lower HbA1c, higher FSH, and lower folate levels. The BMD of female patients is mainly related to hormones, while that of male patients is primarily related to microelements.

## Electronic supplementary material

Below is the link to the electronic supplementary material.

Supplementary Material 1

## Data Availability

The datasets used and analyzed during the current study are available from the corresponding author upon reasonable request.

## Abbreviations

BMI: Body mass index
CPZ: Chlorpromazine
Aps: Antipsychotics
SANS: Scale for the Assessment of Negative Symptoms
SAPS: Scale for the Assessment of Positive Symptoms
Hb: Hemoglobin
SF: Serum ferritin
PLT: Platelet
PTH: Parathyroid hormone
E2: Estradiol
T: Progesterone, testosterone
FSH: Follicle-stimulating hormone
LH: Luteinizing hormone
PRL: Procalcitonin
GLU: Serum glucose
TG: Total triglyceride
HDL: High-density lipoprotein
HbA1c: Glycosylated hemoglobin
CRP: C-reactive protein
FT3: Free triiodothyronine
Ca: Calcium
