# Supplementary material for: Risk factors for osteoporosis in chronic schizophrenia on long-term treatment with antipsychotics: a cross-sectional study
Source: BMC Psychiatry. 2023 Jun 21;23:454. doi: 10.1186/s12888-023-04951-1 (PMC10286449; doi:10.1186/s12888-023-04951-1)
Supplement: Supplementary file 1 — Supplementary Material 1 [file 12888_2023_4951_MOESM1_ESM.docx]

**SUPPLEMENTARY INFORMATION**

**Supplementary Table1.** Gender-based comparisons of demographic and clinical characteristics in patients

| **Characteristic** | **Male** | **Female** | **T / Z / X^2^** | ***P*-value** |
| --- | --- | --- | --- | --- |
|  | **(N=140)** | **(N=71)** |  |  |
| Age | 48.5 (12.3) | 48.7 (11.7) | -0.10 | 0.940 |
| Marital status |  |  | 63.00 | **<0.001** |
| Unmarried | 107 | 15 |  |  |
| Have a spouse | 10 | 29 |  |  |
| Divorced | 21 | 23 |  |  |
| Death of a spouse | 2 | 4 |  |  |
| Education (years) | 7.4 (3.4) | 6.5 (4.1) | -1.60 | 0.100 |
| Body weight (Kg) | 70.2 (12.3) | 62 (11.4) | 4.70 | **<0.001** |
| BMI (kg/m2) | 24.3 (3.5) | 25.5 (5.2) | 1.10 | 0.290 |
| Duration of illness (months) | 273.5 (139.8) | 240.5 (130.4) | 1.70 | 0.100 |
| Smoking (Y/N)^a^ | 53/81 | 1/69 | 34.50 | 0.100 |
| Drinking (Y/N)^a^ | 16/115 | 2/68 | 5.20 | 0.070 |
| Family history of mental illness | 30/110 | 16/55 | 0.30 | 0.720 |
| Family history of metabolic syndrome | 2/138 | 2/69 | 0.50 | 0.490 |
| SANS score | 44.5 (14.1) | 44.7 (17.1) | -0.10 | 0.900 |
| SAPS score | 10 (8.7) | 12.3 (6.6) | -1.90 | 0.060 |
| Antipsychotic CP-dose (g/day) | 0.7 (0.3) | 0.7 (0.4) | 0.20 | 0.880 |
| T-score (degree) |  |  | 4.50 | 0.108 |
| Normal | 84 (60%) | 53 (73%) |  |  |
| Osteopenia | 32 (23%) | 10 (13%) |  |  |
| Osteoporosis | 24 (17%) | 8 (12%) |  |  |
| T-score | -0.82 (1.5) | -0.1 (1.8) | -3.20 | **0.002** |

Abbreviations: BMI, body mass index; CP, chlorpromazine; SANS, Scale for the Assessment of Negative Symptoms; SAPS, Scale for the Assessment of Positive Symptoms (**P*<0.05, ** *P*<0.001).

**Supplementary Table 2.** Correlations between BMD T-score and major variables in patients

| **Variables** | **r** | **p-value** | **Variables** | **r** | ***P*-value** |
| --- | --- | --- | --- | --- | --- |
| Age | -0.343 | **<0.001** | SF | -0.267 | **0.001** |
| Gender | 0.233 | **0.001** | FT3 | 0.010 | 0.085 |
| Drinking | 0.03 | 0.675 | FT4 | 0.061 | 0.377 |
| Smoking | -0.124 | 0.076 | PTH | -0.003 | 0.971 |
| Family history of mental disorder | -0.120 | 0.082 | FSH | 0.225 | 0.425 |
| Education | 0.015 | 0.098 | LH | -0.185 | **0.007** |
| Body weight | 0.042 | 0.542 | E2 | 0.035 | 0.673 |
| BMI | 0.124 | 0.074 | T | -0.190 | **0.006** |
| BMD | 0.826 | **<0.001** | PRL | 0.088 | 0.204 |
| Antipsychotic CP-dose | 0.073 | 0.296 | GLU | -0.160 | **0.021** |
| SANS score | -0.092 | 0.183 | HbA1c | 0.163 | **0.018** |
| SAPS score | 0.155 | **0.025** | BUN | -0.057 | 0.407 |
| Folic acid | 0.203 | **0.01** | CRE | -0.150 | **0.03** |
| Hb | -0.131 | 0.058 | TG | 0.076 | 0.276 |
| 25-OH-VD | 0.114 | 0.1 | HDL | 0.139 | **0.044** |
| Vit-B12 | -0.043 | 0.594 | PLT | 0.120 | 0.083 |

**Supplementary Table 3.** Multiple linear regression analysis of predictive factors associated with the BMD T-score in male and female patients with schizophrenia

|  | **Variables** | **B** | **SE** | **Beta** | **t** | ***P*** |
| --- | --- | --- | --- | --- | --- | --- |
| **Male** **(Adjusted R2 = 0.205)** | Age | -0.03 | 0.01 | -0.256 | -2.76 | 0.007 |
|  | SF | 0.00 | 0.00 | -0.188 | -1.99 | 0.050 |
|  | 25, OH-VD | 0.04 | 0.02 | 0.206 | 2.22 | 0.029 |
|  | PTH | 0.02 | 0.01 | 0.281 | 3.06 | 0.003 |
| **Female (Adjusted R2 =0.426)** | Age | -0.04 | 0.02 | -0.272 | -2.25 | 0.028 |
|  | FSH | -0.02 | 0.01 | -0.321 | -1.87 | 0.066 |
|  | T | 0.24 | 0.11 | 0.205 | 2.15 | 0.035 |
